# Clinical Observation of 108 Cases of Typhoid Fever

**Published:** 1905-03

**Authors:** 


### CLINICAL OBSERVATION OF 108 CASES OF TYPHOID FEVER.

R. M. Harbin (*Medical Record*, November 12, 1904) discusses the treatment of typhoid fever and lays especial stress on restriction of the food given. Surplus food causes accumulations in the intestines, which augment the toxemia, cause diarrhea and increase the risk of hemorrhage. The author employs cocoa, coffee, egg albumen, peptonoids and broths of beef, mutton, chicken, oysters, etc., in prescribed amounts. Milk is undesirable, unless diluted two to five times with one of the other fluids owing to its tendency to form curds. Some of the author's conclusions are that: There is a greater degree of toxemia in cases associated with gastrointestinal disturbances. Emaciation occurs independently of the amount of food taken. A great majority of clinicians of long experience feed typhoid patients in a very restricted manner. Fasting and a restricted diet are indicated, because of pathological conditions, and to a great extent eliminate the dangers of sthenic cases. The proper food management of the case will well-nigh shield the patient from every danger incident to the course of the disease. The eliminative treatment, so-called, is irrational, except to mitigate the sins of overfeeding. What the patient loses in food is more than compensated by a lessened degree of toxemia. Peristalsis favors the absorption of toxins, and cathartics should be used only to remove undigested food. Nitrogenized food and water are essential to a normal nitrogen equilibrium, and there is little danger from inanition in sthenic cases. The presence of ulcers in the intestines should be assumed in every case of typhoid fever, and the proper treatment is rest, which is better attained by fasting and a restricted diet, thus preventing hemorrhage and perforation. The presence of diarrhea and vomiting indicates the adoption of fasting. Fasting and a restricted diet enhance the effects of hydrotherapy and many cases (25 per cent.) run an abortive course. Gelatin is a valuable adjuvant in the dietetics of typhoid fever, and lessens the nitrogenized waste and prevents hemorrhage.—*St. Louis Medical Review.*

